# Ultraviolet, Did the Cell See It from the Side or the Bottom? Assessment and Modeling of UV Effects on Cultured Cells Using the CL-1000 UV-Crosslinker

**DOI:** 10.3390/biotech13040044

**Published:** 2024-10-25

**Authors:** Takahiro Oyama, Kai Yanagihara, Anna Arai, Takanori Kamiya, Midori Oyama, Takashi Tanikawa, Takehiko Abe, Tomomi Hatanaka

**Affiliations:** 1 Hinoki Shinyaku Co., Ltd., 9–6 Nibancho, Chiyoda-ku, Tokyo 102–0084, Japan; 2 Faculty of Pharmacy and Pharmaceutical Sciences, Josai University, 1-1 Keyakidai, Sakado 350-0295, Saitama, Japanoyamami@josai.ac.jp (M.O.); tanikawa@josai.ac.jp (T.T.); tmmhtnk@josai.ac.jp (T.H.); 3 School of Medicine, Tokai University, 143 Shimokasuya, Isehara 259-1193, Kanagawa, Japan

**Keywords:** ultraviolet, model, keratinocytes, crosslinker, NHEK, interleukin-6, dose calculation

## Abstract

Numerous natural extracts and compounds have been evaluated for their ability to mitigate the adverse effects of ultraviolet (UV) overexposure. However, variability in the UV doses that trigger biological responses across studies likely arises from inconsistencies in UV exposure standardization. We hypothesize that these discrepancies are due to variations in culture plates and dishes. The UV dose (D) required to reduce cell viability by 50% differed by a factor of ten between 3.5 cm dishes and 96-well plates. Similarly, the EC_50_ dose for IL-6 release (*D*_1/2_) varied, potentially correlating with the surface area (S). UV exposure to wells with increasing height in 3.5 cm dishes resulted in a decrease in IL-6 release, suggesting that the greater the well height, the more it may influence UV exposure through reflection or shielding effects, thereby contributing to the physiological effects on the cells. To compare these differences among plates, we defined the height-to-diameter ratio (r). Analysis revealed a linear correlation between *D*_1/2_ and S in a log-log plot, and between *D*_1/2_ and r in a semi-log plot. From this, we defined two empirical indices *σ* and ρ for UV dose adjustment. A deductive model was also developed to derive a D′ value that adjusts UV doses without requiring training. As with σ and ρ, the UV dose D was effectively adjusted using D′ as well. These attempts suggest that D′ offers a foundational framework for evaluating UVB effects on cultured cells.

## 1. Introduction

Exposure to ultraviolet (UV) radiation is an unavoidable aspect of life [1]. Recently, its detrimental effects have gained attention from both medical and cosmetic perspectives [2]. The skin, which is the outermost barrier, protects underlying organs from UV radiation. UV exposure primarily induces the formation of reactive oxygen species (ROS) [3], DNA damage [4], apoptosis [5], and damage to the extracellular matrix (ECM) [6] in keratinocytes. These keratinocytes then release inflammatory cytokines, such as interleukin (IL)-6, IL-1α, IL-1β, and tumor necrosis factor-alpha (TNF-α) [7]. Such damage can lead to skin carcinogenesis and photoaging [2,8,9]. To mitigate these adverse effects, various materials, particularly plant extracts and natural compounds, have been evaluated for their ability to prevent UV-induced damage [10,11].

**Figure 1:**
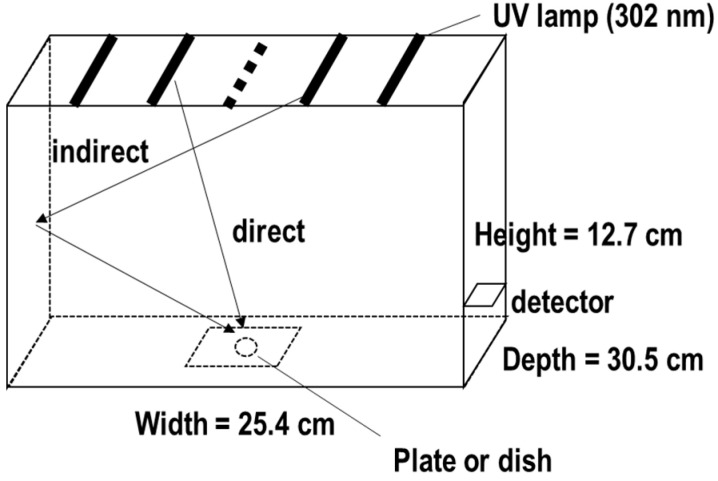
Specifications of the CL-1000 UV Crosslinker. The dimensions of this equipment are width = 25.4 cm, depth = 30.5 cm, and height = 12.7 cm. The equipment accommodates up to five fluorescent tubes with UVB emission at 302 nm. The dotted line indicates a UV lamp that was detached in this study. The inner walls of the equipment can reflect UV radiation, allowing plates to receive UVB both directly and indirectly. A detector located at the inner back right side of the equipment monitors the exposure automatically once the UV dose reaches the preset value.

The physical properties of UV radiation involve photons that inherently carry energy [12]. The radiant flux of UVB is quantified as Φ_e_ [W (J/s)], which is related to the electrical power consumed by the UV tube, and is reduced by the conversion efficiency. UV sensors typically measure irradiance, expressed as I_R_ [W/m^2^ (J/s/m^2^)]. Integrating I_R_ over time yields the fluence [J/m^2^]. In biological assays, fluence, typically expressed in units of mJ/cm^2^ or J/m^2^, is used to standardize UV exposure, which is referred to as the UV dose (D) [13]. In UV biological evaluations, researchers generally employ one of two approaches: 1. constructing their own equipment using UV tube lamps and regulating D in combination with a UV detector [14], or 2. utilizing UV crosslinkers, such as the CL-1000/3000 series by Analytik Jena US [15,16,17,18,19,20] or the BLX-Multichannel Bio-Link crosslinker by VILBER LOUMAT [21], and others. Both types of equipment operate on a similar principle. Here, we discuss the CL-1000 model (Figure 1). The dimensions of this equipment are width = 25.4 cm, depth = 30.5 cm, and height = 12.7 cm. According to the manufacturer, users can select wavelength from three options: 365 nm (UVA), 302 nm (UVB), and 254 nm (UVC). The equipment can accommodate up to five fluorescent tubes. The inner walls can reflect UV radiation, thereby allowing efficient UV exposure to the samples through both direct and indirect means. A detector located at the inner back right side of the equipment halts the exposure automatically once the UV dose reaches the preset value.

Using this equipment, researchers have demonstrated that various biological events occur due to UVB exposure across different cell types (Table 1). However, we observed discrepancies in the UV doses used in these studies. We hypothesized that the UV dose set in the instrument required to elicit a similar response in cultured cells varies with the type of culture plate. In this study, we aimed to demonstrate clearly the difference in UV-induced changes of cell viability properties and inflammatory response among cell culture plates and dishes. Consequently, we worked on establishing indices for correcting the UV dose through both empirical and theoretical approaches.

## 2. Materials and Methods

### 2.1. Cells and Cell Culture

Normal human epidermal keratinocytes (NHEKs) were purchased from Kurabo (Osaka, Japan) and cultured in a specialized medium (HuMedia-KG2) from Kurabo. NHEK cells are the primary culture of neonatal human keratinocytes. The purchased cells were thawed and pre-cultured at 37 °C in a humidified 5% CO_2_ atmosphere in a standard cell culture flask (Sumitomo Bakelite Co., Ltd., Tokyo, Japan). The cells used for the study were limited to days 5–19 post-arrival. The culture medium contains several supplements, including 0.1 μg/mL human EGF, 0.67 mg/mL hydrocortisone, 10 mg/mL insulin, 50 mg/mL gentamicin, 50 μg/mL amphotericin B, and 2 mL of bovine pituitary gland extract (BPE) in 500 mL HuMedia-KG2, as per the manufacturer’s instructions. Two days prior to the start of the experiment, cells were trypsinized and subcultured into 96-, 24-, and 12-well plates, as well as 3.5 cm dishes, under the conditions listed in Table 2.

### 2.2. UV Exposure

UV irradiation was performed using a CL-1000M (Analytik Jena US, an Endress + Hauser Company, Upland, CA, USA) with a 302 nm, 8 W UV tube. To adjust the UV intensity, 4 out of 5 tubes were used as illustrated in Figure 1. Culture plates were placed at the center of the equipment. The UV doses were set at 2, 5, 10, 20, 30, 50, 100, 200, and 500 mJ/cm^2^, according to the experimental requirements.

### 2.3. Trypan Blue Dye Exclusion Assay

The culture medium from 3.5 cm dishes was centrifuged at 4 °C, 5000× *g*. The supernatant was used for ELISA (Section 2.6). The dishes were rinsed with 1 mL of PBS, which was then mixed with the precipitate of the medium (floating cell fraction). Subsequently, 0.5 mL of accutase (NACALAI TESQUE, INC., Kyoto, Japan) was added to detach cells in a 37 °C incubator for 5 min. The cell suspension was mixed with the floating cell fraction and centrifuged again at 4 °C, 5000× *g*. The precipitate was resuspended in PBS. The cell suspensions were mixed with 0.4% *v/v* trypan blue dye (Fujifilm Wako Pure Chemical Corporation, Osaka, Japan) in a 1:1 ratio, and cells were immediately counted using an improved Neubauer cell counter (WASTON Co., Ltd., Tokyo, Japan) under microscopic observation with an EVOS XL Core Imaging System (Thermo Fisher Scientific Inc., Waltham, MA, USA).

### 2.4. Hoechst 33342 Staining

The Cell Count Normalization Kit (DOJINDO LABORATORIES, Kumamoto, Japan) was used according to the manufacturer’s instructions with slight modifications. The cultured cells in 96-well plates were rinsed with Hank’s Balanced Salt Solution (HBSS) (+) (Fujifilm Wako Pure Chemical Corporation) and stained using the kit. After staining, the wells were rinsed with HBSS (+) twice and replaced with 100 μL of fresh HBSS (+). Fluorescence was measured at Ex/Em = 351/461 nm using a SYNERGY H1 multimodal plate reader (Agilent Technologies, Inc., Santa Clara, CA, USA). Data were expressed as relative values against the non-UV-exposed groups.

### 2.5. WST-8 Assay

WST-8 reduction activity was assessed using the Cell Counting Kit-8 (DOJINDO LABORATORIES) according to the manufacturer’s instructions. Absorbance was measured at 450 nm using a SYNERGY H1 multimodal plate reader. Data were expressed as relative values against the non-UV-exposed groups.

### 2.6. ELISA

The ELISA MAX™ Deluxe Set Human IL-6 (BioLegend, Inc., San Diego, CA, USA) was used according to the manufacturer’s instructions. Culture media from 96-, 24-, and 12-well plates were used directly. Media from the 3.5 cm dish group were collected during the procedure described in Section 2.3 (Trypan Blue Dye Exclusion Assay). Absorbance was measured at 450 nm using a SYNERGY H1 multimodal plate reader.

### 2.7. Curve Fitting

A 4-parameter sigmoid curve was used as the target model [22].

(1) $$y=d+\frac{a-d}{1+{\left(\frac{x}{c}\right)}^{b}}$$

where *y* is the response and *x* is the dose. Here, parameter *a* represents the bottom of the curve or lower plateau, and *d* represents the upper plateau. The steepness of the linear portion of the curve is described by the slope factor, *b*. Parameter *c* is the concentration corresponding to the response midway between *a* and *d*. The formulas and parameters were defined using the “def” function in Python. The “curve_fit” function of the “scipy.optimize” module in Python was used to estimate all parameters, utilizing the nonlinear least squares method for curve fitting [23]. The program was run on Google Collaboratory with the runtime set to “Python 3” (Supplementary Material S1).

The least squares method programmed in Microsoft Excel 2021 (Microsoft Corporation, Redmond, WA, USA) was used for a linear regression. 

### 2.8. Measurement of Transmittance (%T)

The transmittance of HuMedia-KG2 was measured using a UV-1280 UV-VIS spectrometer (SHIMADZU CORPORATION, Kyoto, Japan). One milliliter of HuMedia-KG2 was transferred into disposable UV cuvettes UVC-SM (AS ONE Corporation, Osaka, Japan), and absorbance at 302 nm (*A*) was recorded. Transmittance coefficient (*T*) was calculated using the following equation:

(2) $$T=\frac{\%T}{100}=\frac{10^{2-A}}{100}=10^{-A}$$

Samples were diluted by half three times. The relative concentration, standardized against the original sample, is expected to correlate with concentration according to the Lambert-Beer’s law:

(3) A=εcl

where *ε* is molar absorption coefficient, *c* is the molar concentration, and *l* is optical path length. The *ε* was calculated from correlation between the relative concentration and absorbance by a linear regression.

### 2.9. Statistical Analyses

Data were corrected using the method of Ruijter et al. [24,25]. All quantitative data are presented as the mean ± standard deviation (SD). Statistical analysis was performed on Google Collaboratory with the runtime set to R (ver. 4.4.0). Statistical tests for differences between multiple comparisons were conducted using Dunnett’s test. The significance level was set at α = 0.05.

### 2.10. Declarations of Generative AI in Scientific Writing

During the preparation of this work, the authors used ChatGPT: model architecture GPT-3.5 (OpenAI, San Francisco, CA, USA) to paraphrase and refine expressions. After using this tool, the authors reviewed and edited the content as needed and take full responsibility for the content of the publication. ChatGPT was also used for preparing Python programs.

## 3. Results

### 3.1. Analysis of Cytotoxic Effects Against UVB Exposure on Different Plate Formats

**Figure 2:**
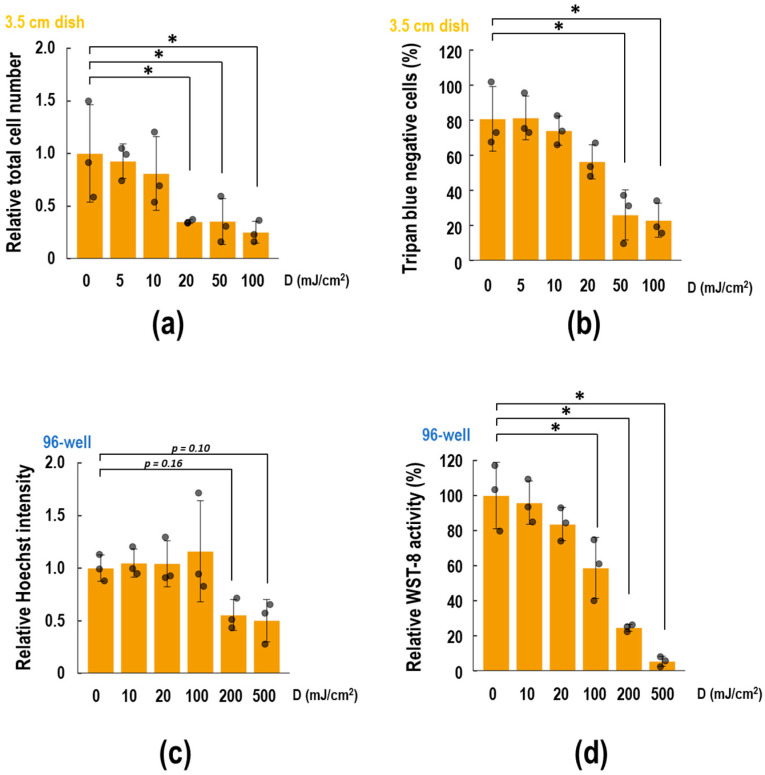
The dose-dependent cytotoxic effects of UVB on cultured keratinocytes. NHEK cells were seeded in both 3.5 cm dishes and 96-well black plates as indicated in Table 2. After a 2-day preculture, the indicated doses of UVB were irradiated to the wells. Cells were subjected to each experiment after an additional 1-day incubation. (**a**,**b**) Cells in 3.5 cm dishes were detached and counted under a microscope, mixed with an equal volume of 0.4% *v*/*v* trypan blue dye. The relative numbers of (**a**) total cells and (**b**) trypan blue-negative cell rates (%) were calculated. (**c**,**d**) Cells in 96-well plates were (**c**) stained with Hoechst 33342 for 30 min or (**d**) incubated with WST-8 reagent for 60 min. Individual quantified values are represented as gray dots on the bar chart. All data are expressed as the mean ± SD of at least three independent experiments. Statistical analyses were performed using Dunnett’s test, compared to the 0 mJ/cm^2^ group. * *p* < 0.05.

First, we compared the cytotoxic effects of UVB exposure on 3.5 cm dishes and 96-well plates. As shown in Figure 2a, UV exposure up to 10 mJ/cm^2^ did not affect the total cell number, whereas exposure to at least 20 mJ/cm^2^ decreased the remaining cells to less than half of the control. The cell death rate was assessed by trypan blue dye exclusion assay in the same samples. Consequently, trypan blue negative (viable) cells decreased in a UV dose-dependent manner (Figure 2b, EC_50_ = 22.2 mJ/cm^2^). In contrast, when cells were cultured in the 96-well plates, the UV dose that reduced the cell number by half was 200 mJ/cm^2^, as indicated by the fluorescence of Hoechst-stained cells (Figure 2c). WST-8 reduction activity of the cells decreased in a UV dose-dependent manner (Figure 2d, EC_50_ = 117.7 mJ/cm^2^). These results suggest that the UV dose affecting cells varies with the plate format.

### 3.2. Analysis of IL-6 Release Against UVB Exposure across Different Plate Formats

**Figure 3:**
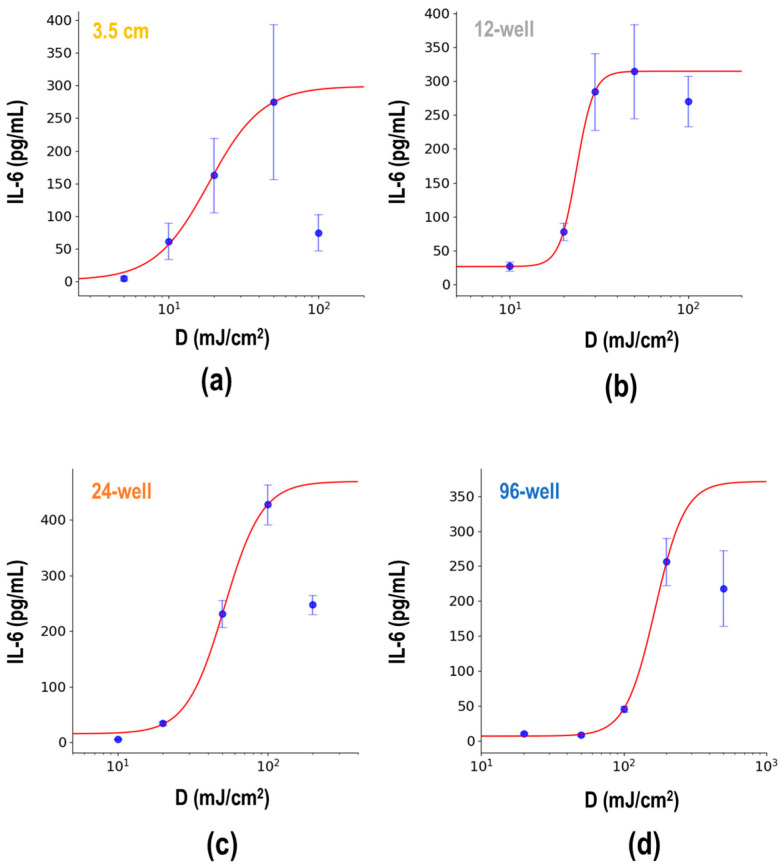
The dose-dependent inflammatory effects of UVB on cultured keratinocytes. NHEK cells were seeded in (**a**) 3.5 cm dishes, (**b**) 12-well, (**c**) 24-well, and (**d**) 96-well plates as indicated in Table 2. After a 2-day preculture, the indicated doses of UVB were irradiated to the wells. Following a 24 h incubation, IL-6 concentrations in the supernatant medium were measured by ELISA. All data are expressed as the mean ± SD of at least three independent experiments (blue dots). Data excluding the highest dose group were fitted with a 4-parameter sigmoid curve, indicated by the red line. The parameters predicted by the calculation are shown in Table 3.

To elucidate the difference in plate formats in detail, we compared the IL-6 release levels in 3.5 cm, 12-well, 24-well, and 96-well plates and dishes. As a result, IL-6 release increased in a UV dose-dependent manner, but beyond a certain dose, the IL-6 release level decreased in all formats (Figure 3). To determine a pattern for EC_50_, data excluding the decreased points were subjected to 4-parameter sigmoid curve fitting (Table 3). The estimated *c* value represents the UV dose causing half the level of IL-6 release, defined as *D*_1/2_. These results suggest that the UV dose that causes the same inflammatory response is different in the surface area of culture plates and dishes.

### 3.3. Effect of Equipment Configuration on the IL-6 Release

**Figure 4:**
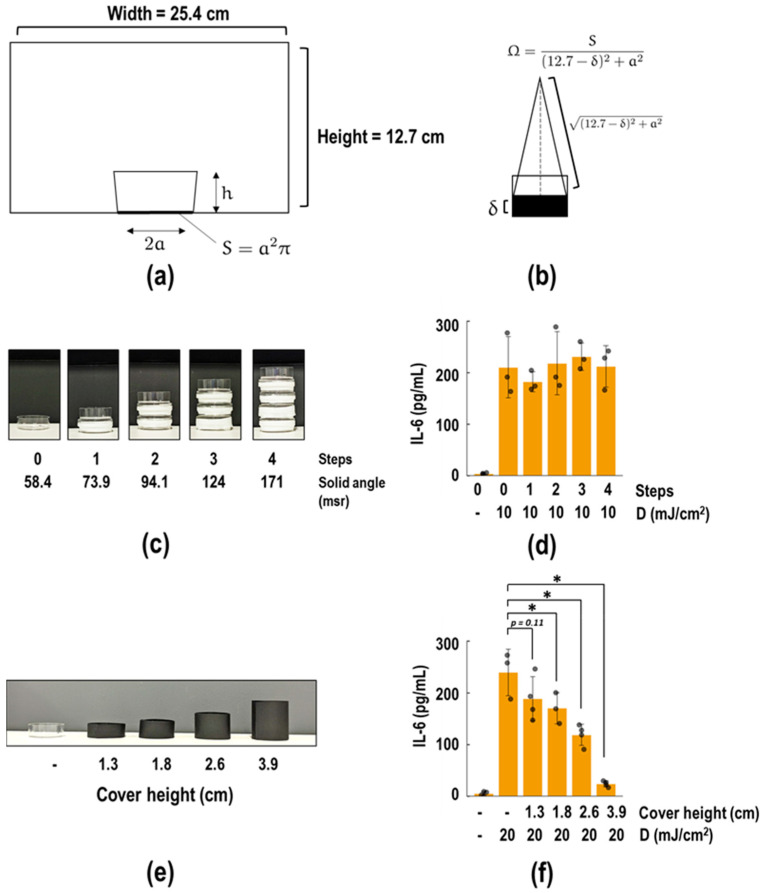
Investigation of the change in IL-6 release based on the solid angle and height of the well wall. (**a**) Cross-sectional view from the side of the CL-1000. A well placed in the center of the equipment is schematically depicted. The height and radius of the well are expressed as h and a cm, respectively. (**b**) The conceptual drawing of the calculation of the solid angle in the case of stacking dishes. (**c**) Pictures of stacking dishes. (**d**) The 5.0 × 10^4^ NHEK cells were seeded in 3.5 cm dishes under the conditions indicated in (**c**). After a 2-day preculture, 10 mJ/cm^2^ of UVB was irradiated to the wells. Following a 24 h incubation, IL-6 concentrations in the supernatant medium were measured by ELISA. (**e**) Pictures of covering dishes with a cylinder made of black paper. (**f**) The 5.0 × 10^4^ NHEK cells were seeded in 3.5 cm dishes under the conditions indicated in (**e**). After a 2-day preculture, 20 mJ/cm^2^ of UVB was irradiated to the wells. Following a 24 h incubation, IL-6 concentrations in the supernatant medium were measured by ELISA. Individual quantified values are represented as gray dots on the bar chart. All data were expressed as the mean ± SD of at least three independent experiments. Statistical analyses were performed using Dunnett’s test, compared to the 1.3 cm-covered group (**g**). * *p* < 0.05.

To elucidate the variation in biological effects of UVB dose with the surface area of culture plates, we investigated the relation between these effects and solid angles. Assuming that a point light source (A) at the center of the ceiling represents all UVB light, forming a cone with vertex A and base S, the solid angle Ω was calculated as

(4) $$\Omega =\frac{S}{{\left(12.7-\delta \right)}^{2}+a^{2}}$$

where δ is the height from the bottom of the equipment (Figure 4a,b). The solid angles for 96-, 24-, 12-well plates and a 3.5 cm dish are 2.17, 11.7, 23.4, and 58.4 msr, respectively. By stacking dishes to reduce the distance to A, the solid angles increase (Figure 4c). The stacking of dishes 1, 2, 3, and 4 results in solid angles of 73.9, 94.1, 124, and 171 msr, respectively. As shown in Figure 4d, IL-6 release remained unchanged by the distance from the ceiling, for exposure to 10 mJ/cm^2^ UVB. This experiment rejected the assumption of a point light source. We then considered that a larger S might receive more oblique light due to its increased surface area. We assessed whether covering the dish with a black paper cylinder (Figure 4e) could prevent UV exposure in this system. A 1.3 cm cover, the same height as the original 3.5 cm dish, reduced IL-6 release by 24.8%; however, this reduction was not significant (Figure 4f; *p* = 0.1057). As expected, taller paper cylinders further reduced IL-6 release (Figure 4f). These results suggest that the UVB effects on cultured cells are not influenced by the distance from the UV lamp but are affected by the height of the well wall.

### 3.4. Development of Empirical Formula

**Figure 5:**
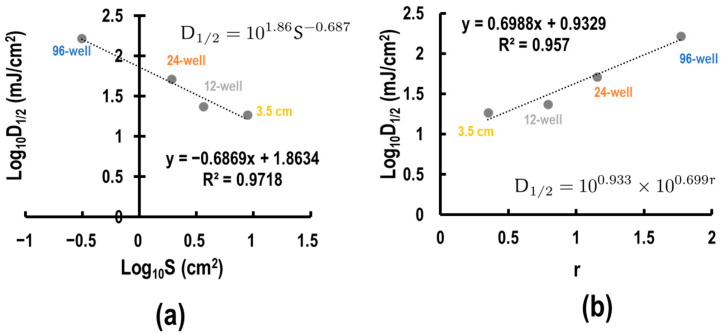
Correlation between S and *D*_1/2_, and between r and *D*_1/2_. (**a**) Scatter plot between log_10_(S) and log_10_(*D*_1/2_) with a regression line described by the formula y = −0.6869x + 1.8634. (**b**) The scatter plot between r and log_10_(*D*_1/2_) with a regression line described by the formula y = 0.6988x + 0.9329.

Considering our data together, we found two relationships between the effective UVB dose and variables related to the well format, which was determined by the variables of the surface area *S* and the height of the wall *h*. To elucidate relationships, we applied linear regression analyses of various combinations of the variables. Upon analysis, we discovered that *D*_1/2_ and S were linear in the log-log plot (Figure 5a) with R^2^ = 0.971. This led to the formula *D*_1/2_ S^0.687^ = Const. Assuming that this relationship between *D*_1/2_ and *S* is generalizable, *σ*, representing the corrected UV index, can be defined as follows:

(5) $$\sigma =DS^{0.687}$$

using this equation, the EC_50_ values for the WST-8 assay (96-well plate, 117.7 mJ/cm^2^) and the trypan blue assay (3.5 cm dish, 22.2 mJ/cm^2^) were adjusted to σ = 52.84 and 99.41 mJ/cm^−1.374^, respectively. 

Contrarily, the data in Figure 4f mean that the oblique UVB light poured into the well is important to consider; in other words, the apparent surface area difference is actually height/diameter ratio (r) difference. The r values for the 96-well, 24-well, 12-well plates, and 3.5 cm dish were 1.770, 1.155, 0.791, and 0.351, respectively (Table 2). We assessed the relationship between r and *D*_1/2_. Upon analyses, *r* and *D*_1/2_ were correlated in the semi-log plot (Figure 5b) with R^2^ = 0.957. Assuming that this relationship between *D*_1/2_ and *r* can be generalized, ρ, representing the corrected UV dose, can be defined as follows:

(6) $$\varrho =\frac{D}{5.0^{r}}$$

Using this equation, the EC_50_ values for the WST-8 assay (96-well plate) and the trypan blue assay (3.5 cm dish) were adjusted to ρ = 6.81 and 12.61 mJ/cm^2^, respectively.

### 3.5. Calculation of the Absorptivity and Transmittance Rate

**Figure 6:**
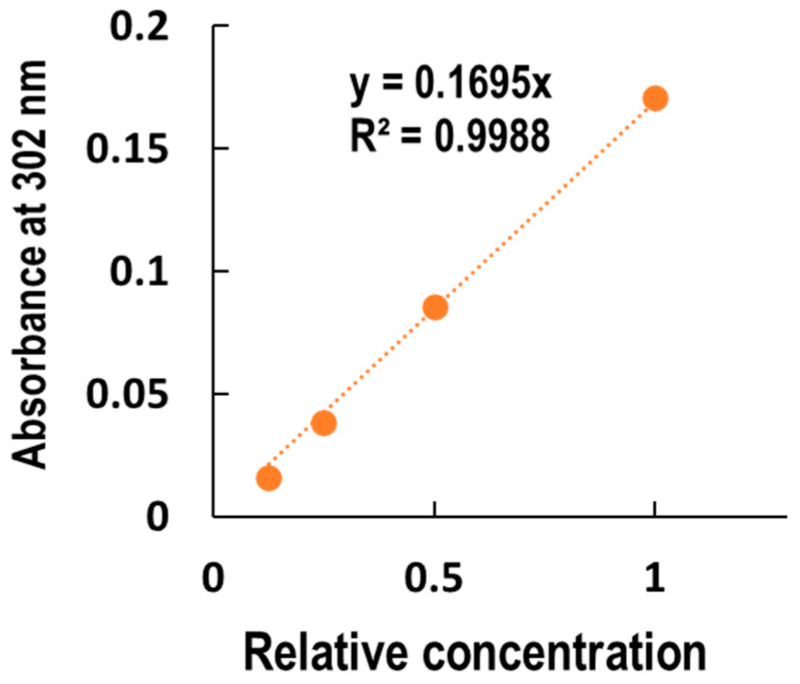
Absorbance change with the serial dilutions of HuMedia-KG2. The medium specialized for keratinocytes (HuMedia-KG2) was serially diluted and absorbance measured at 302 nm. Since the molar concentration was not defined, the relative concentration of the undiluted solution was taken as 1. The data were fitted with a linear regression, setting the intercept to 0.

To obtain absorptivity *ε* of the media, the absorbances after serial dilution of the medium were measured (Figure 6). The concentration of the solutions was defined as the relative values of the undiluted solution. From the Lambert–Beer law (Equations (2) and (3)), the absorptivity ε and transmittance rate %T of undiluted media were calculated to be 0.1695 and 89.0%, respectively.

### 3.6. Comparison of the Adjustment of UV Dose by Indices σ, ρ and Model D′

**Figure 7:**
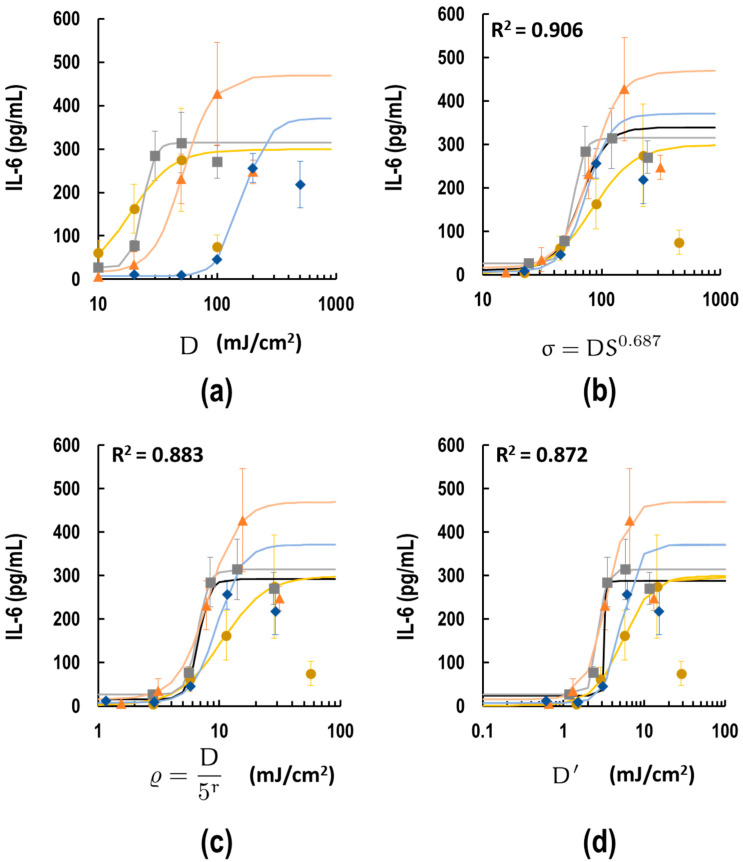
Comparison of the corrected dose–effect plot using three indices as dose. (**a**) IL-6 release by UVB exposure from Figure 4 re-plotted in one figure. (**b**–**d**) IL-6 response curves using (**b**) *σ*, (**c**) ρ, and (**d**) D′ as the *X*-axis. Data for 3.5 cm dish, 12-well, 24-well, and 96-well plates are represented as mustard circles, gray squares, orange triangles, and blue diamonds, respectively. Each dataset was fitted with a 4-parameter sigmoid curve shown by the smooth lines with same colors. All data are expressed as mean ± SD. Data from all plates were combined and fitted with a 4-parameter sigmoid curve, depicted by the black line. The R^2^ values of the fittings for all values are shown in the upper left of each graph.

**Figure A1:**
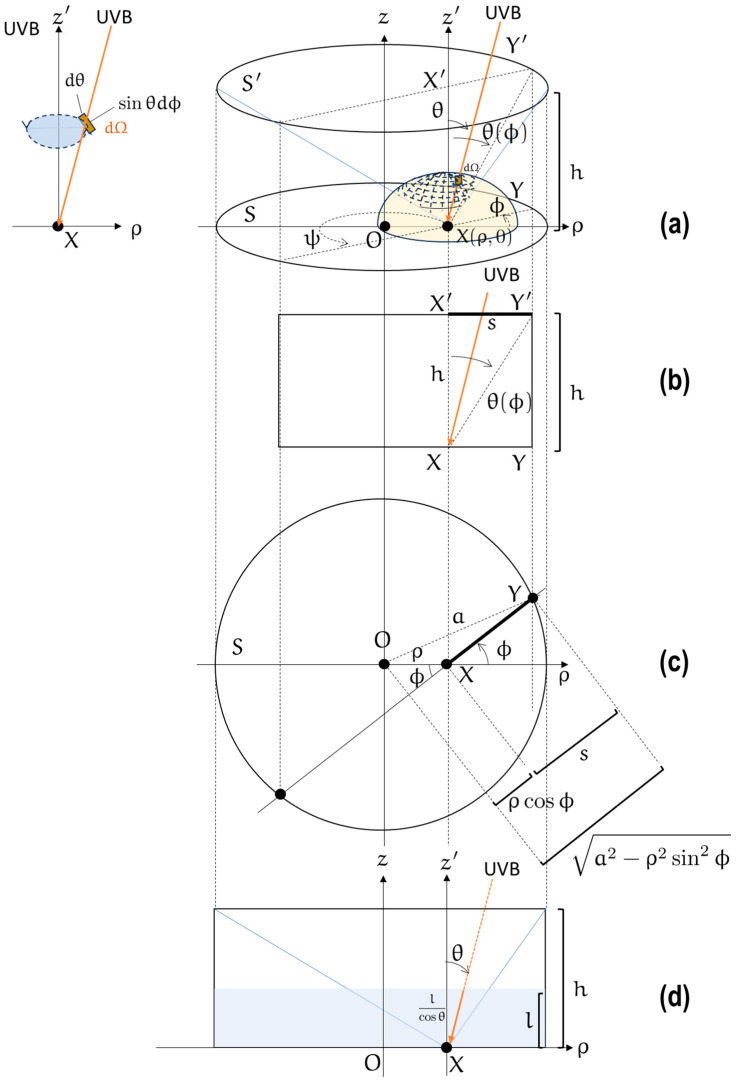
Schematic diagram of wells on culture plates and dishes. A well is placed in the ρψz -space with the center of its bottom surface at origin O. (**a**) Spherical coordinates placed in a well. As all UVB rays pass through the corresponding surface of the hemisphere, the dose at point X is calculated by integrating over spherical coordinates. The z’-axis is parallel to *z*-axis passing through X. Left panel shows an illustration of area element calculation. The area element dΩ=sin⁡θdθdϕ. (**b**) A cross-section of a well cut by a plane passing through a specific point X and inclined at an angle φ. An intersection point of the circle O and the plane directed to ρ>0 is named Y. s is the length of XY. (**c**) A top view of the well. (**d**) Cross-section of a well cut by the ρz-plane. a, radius of the well; h, height of the well; l, height of the filled medium.

Since the indices *σ* (Equation (5)) and ρ (Equation (6)) are just empirical expressions whose parameters can be easily changed under the measurement conditions, we attempted to construct a model by the geometrical consideration (Appendix A), and established adjustment index D′ theoretically. The modeling concept focuses on a specific coordinate on S, X(x, ψ), while considering all rays converging at that point (Figure A1). In this model, UVB rays originate from all directions surrounding the well due to reflections from the equipment’s walls. Assuming that the well walls do not transmit UVB rays, the UV dose reaching X is constrained by the height of the wells. Additionally, the culture medium attenuates the UVB rays, necessitating the application of a reduction factor to D. The calculated dose at X is denoted as z_E_(x) in this model. Subsequently, the z_E_(x) values were averaged over S, yielding a representative dose defined as the D′ value. After all, three indices for adjusting UV dose variations across different culture plates were compared. Here, the raw data were replotted on the same graph with the dose axis in D (mJ/cm^2^) (Figure 7a). Next, doses at each point were converted by *σ* (Figure 7b), ρ (Figure 7c), and D′ (Figure 7d). The adjustment by *σ* and ρ consolidated the four sigmoid curves representing each plate and dish into a unified index. This result could be explained by the fact that the two empirical indices are set to match the EC_50_ values. Fitting the consolidated data from the four plates and dishes, indicated as black lines, showed good fits for *σ* (R^2^ = 0.906) and ρ (R^2^ = 0.883). Additionally, using D′ as the *X*-axis also showed good fits for the data (R^2^ = 0.872) (Figure 7d). The EC_50_ value for D′ (3.19 mJ/cm^2^) was the same order as that of ρ (6.76 mJ/cm^2^) but differs from that of *σ* (66.50). These results suggest that the model constructed in this study fits the real data and is aligned with the empirical equation.

## 4. Discussion

Ultraviolet radiation has harmful effects on the body, including carcinogenesis and aging [9]. To mitigate these effects, many natural compounds have been assessed for their ability to prevent cell death or inflammation both in vitro and in vivo, to apply them to sunscreen formulations [26]. In many of these UV protection assessments, the CL-1000/3000 UV crosslinker is frequently used (Table 1). This UV crosslinker is equipped with detectors and is programmed to stop once the desired dose of irradiation is reached. Thus, researchers can consistently regulate each irradiation dose to the targeted level throughout the experiment. However, we noticed that the reported UV doses for assessment vary in the literature.

In this study, we focused on how UVB exposure dose varies with the scale of culture plates. As expected from the literature search (Table 1), cell viability and IL-6 release varied across different types of cell culture plates (Figure 2 and Figure 3). Moreover, the analysis of the EC_50_ dose of IL-6 release revealed that the effect of UV depends on the culture plate surface area (Figure 3). We obtained two empirical indices and also constructed one model. The index *σ* could simply adjust the dose difference using only the surface area parameter S. As shown in Figure 4e,f, the difference in UV dose by the type of plate was also affected by the height of the well. However, among the indices, *σ*, which does not use height information, is the best fit (R^2^ = 0.906), presumably because most culture plates and dishes are standardized across equipment like plate readers, and thus are considered to have the same height. However, *σ* has an unusual formal dimension, mJ cm^−1.374^. Conversely, the index ρ accounts for two parameters, diameter 2a and height h. The model fit is moderate (R^2^ = 0.883), but it can account for more generalized culture wells including height information. Moreover, the dimension of ρ is mJ/cm^2^, which can be more advantageous than *σ* at this point. The difference in dimension reflects the difference in EC_50_ value estimation using both indices. The EC_50_ value of *σ* (66.50) was ten times larger than that of ρ (6.76 mJ/cm^2^), indicating that the *σ*-adjusted dose is overestimated by a factor of cm^1.374^. This is because σ and ρ were not standardized by the proportionality factors. Ideally, the indices σ and ρ should be expressed as follows:

(7) $$\sigma =k_{1}DS^{0.687}$$

(8) $$\varrho =k_{2}\frac{D}{5.0^{r}}$$

where *k*_1_ has the dimension cm^−1.374^, and *k*_2_ is a unitless factor. Determining these factors is beyond our current understanding, as it is an issue related to regulation and standardization. For instance, if we define *k*_1_ such that the σ_3.5 cm dish_ equals D, then *k*_1_ would be calculated as 1/8.87^0.687^ = 0.223 cm^−1.374^. Another pattern is considering a sufficiently large S. The maximum radius that can be placed in the equipment is 25.4 cm, which corresponds to the width of the equipment. In this situation, *k*_1_ should be calculated to 0.014. Considering simply the formula (7), as S approaches infinity, σ also diverges to infinity. This suggests the formula (7) only fits in the narrow range in S. k_2_ can also be defined such that ρ_3.5 cm dish_= D (*k*_2_ = 1.759). Meanwhile, if r reaches 0 (h → 0), ρ approaches *k*_2_D, which perhaps means the necessity to set *k*_2_ = 1. In the future, we expect advancements in the standardization and metrology of this phenomenon.

Another disadvantage of these two indices is that the constants and coefficients of these formulas are not fixed because they are empirical expressions. It is possible that they may change if the conditions or analytes change. Therefore, we developed a model that represents the total amount of UVB light absorbed by the wells. This model was developed based on geometric principles and includes parameters such as radius *a*, well height *h*, medium volume *v*, and the medium’s absorption coefficient ε (Supplementary Materials S2). The model can calculate the actual UVB energy received by each cell culture plate without the need for training on real data. As shown in Figure 7d. the dose adjusted by D′ is the same order as that corrected by ρ, which is an empirical expression. However, the model fit coefficient R^2^ = 0.872 was lower than those of the empirical equations (Figure 7). This suggests that further refinement is needed in the model. The limitations of the model include the following: 1. UV light attenuation due to reflection by the equipment walls, 2. the assumption that the liquid surface is horizontal without accounting for surface tension, 3. the refraction of the radiation in the passage from air to medium being ignored, 4. the assumption that the transmittance and reflection rate through the plastic walls of cell culture plates is 0%. Actually, experiments using a 3.5 cm dish covered with a black paper cylinder of the same height showed a 24% decrease in IL-6 release, though this was not significant (Figure 4e). 5. The model is not compatible with the floating cell culture. 

Currently, no widely accepted generalized and standardized machine and culture wells for UV irradiation have been developed, and researchers often construct their own systems, typically using tube-based UVB light sources [14]. This setup inevitably results in a difference in the amount of diagonal light exposure, influenced by r values, causing dose differences between containers as well, like using the commercial UV crosslinker shown in this study. Future work should focus on refining the model and/or developing new equipment that ensures uniform UV dosing across cell cultures. Until a precise model is developed in the future, researchers can use *σ* or ρ, especially within the range of 0.31 ≤ S ≤ 8.87, to convert the UV dose. For standardization, medium volume and cell density are also important factors. In this study, the height of the culture medium (l) and cell density were not consistent across the four plates, as shown in Table 1, because our data included preliminary trials. This is an obstacle to estimating precise coefficients with large R^2^ values in each model. The standardization or correction of these factors also needs to be considered. 

The WST-8 assay is commonly used for cell viability assessment [27]. The method depends on a chemical whose absorption wavelength changes with its redox state. Specifically, the reduced WST-8 dye absorbs at 450 nm, appearing orange and indicating viable cells. The trypan blue assay is also used for cell viability testing [28]. This method uses a membrane-impermeable dye. When the membrane integrity is disrupted in dead cells, the dye permeates, resulting in blue staining. Thus, the ratio of blue-stained cells indicates the cell death rate. Although these assays are used broadly for testing cell viability, it has been suggested that these methods do not necessarily represent cell death in certain situations [29,30]. Consequently, researchers often combine these methods to assess the effects of stimuli more comprehensively. However, interpreting results from UV-induced studies, especially when using a CL-1000 UV cross-linker, is complex due to biological factors and scale considerations. Researchers select experimental scales according to objectives, cost, and practicality. For example, while the WST-8 assay theoretically applies across all scales, its cost-effectiveness is optimized in smaller scales such as 24-, 96-, and 384-well formats. In contrast, trypan blue staining needs cell counting under microscopy, requiring at least 4.0 × 10^5^ cells/mL. In this study, WST-8 and trypan blue assays were conducted using a 96-well plate and a 3.5 cm dish, respectively (Figure 2). Raw EC_50_ data from WST-8 and trypan blue assays were 22.2 and 117.7 mJ/cm^2^, respectively. Honest interpretation reveals cell death events occurring at lower UV doses than those affecting redox states. As discussed earlier, misinterpretation due to scale factors can make such conclusions inaccurate.

To account for differences among culture scales, we established an equation that relates surface area to biologically active UV dose (Equations (5) and (6)). Here, we consider the index *σ*, the most fitted index in this study. After adjustment with *σ*, the EC_50_ doses for WST-8 and trypan blue assays were 50.67 and 95.07, respectively. This provides a more reasoned explanation for why the UV dose affecting cellular redox state was smaller than that inducing cell death. Total cell counting and Hoechst staining indicated that UV doses stopping NHEK cell proliferation ranged from 10 to 20 and from 100 to 200 mJ/cm^2^, respectively (Figure 2). After consolidation by index *σ*, these ranges were adjusted to 44.8 to 89.6 and 44.9 to 89.8, respectively. The EC_50_ value for UV-induced IL-6 release was 10^1.86^, which is equal to *σ* = 72.4 (Figure 5a). In summary, UV exposure initially impacts cellular redox systems (*σ* = 50.67), followed by proliferation (*σ* = 44.8 to 89.8), inflammatory cytokine secretion (*σ* = 72.4), and eventually leading to cell death (*σ* = 95.07). This approach enables comparison of UV effects across various evaluation metrics by mitigating the influence of culture plate scale. 

## 5. Conclusions

The UVB amount received by cultured cells varies with the type of cell culture plate, primarily because the well walls influence the amount of diagonal light exposure. The height-to-diameter ratio r is a critical variable. The empirical formula ρ effectively adjusted differences among plates. If the well wall is constant, another experimental formula *σ*, which depends on surface area S rather than r, provides a more accurate adjustment for UV dose. Additionally, we developed a model to calculate the actual UV dose without the need for training data. It provides a preliminary attempt toward developing a more precise model for evaluating UVB effects on cultured cells. We recommend specifying the type of culture plate used in UV exposure experiments and writing down *σ*, ρ, or D′ values along with D if possible.

## Figures and Tables

**Table 1:** Experimental conditions in literature using CL-1000.

Cells	UV Dose (mJ/cm^2^)	Biological Events	Culture Plate	Author	Ref.
HaCaT	150	44% of cell viability	24-well	Kim et al.	[16]
NHEK	120	Intracellular ROS generation	96-well plate	Lin et al.	[17]
NHEK/ HaCaT	<100	Confirmation of not showing decrease in cell viability	96-well plate	Ohnishi et al.	[18]
HaCaT	80	No effect on cell survival	6 cm dish	Xuan et al.	[19]
		MMP-1 expression			
NHEK/ HaCaT	50	Reduction in CXCL10 expression	N. D. ^1^	Ohnishi et al.	[18]
Hs68	40	MMP-1 expression	10 cm dish	Chiang et al.	[20]
HaCaT	30	MMP-1 expression	N. D. ^1^	Song et al.	[15]

HaCaT, spontaneously transformed aneuploid immortal keratinocyte cell line from adult human skin; NHEK, normal human epidermal keratinocyte; Hs68, human foreskin fibroblasts. ^1^ Not described in the source.

**Table 2:** The profiles of cell culture plates and dishes in this study.

	96-Well	24-Well	12-Well	3.5 cm
Supplier	BM Bio	Falcon	Sumitomo Bakelite Co., Ltd.	Sumitomo Bakelite Co., Ltd.
Catalog No.	215,006	353,047	MS-80120	MS-10530
Well diameter 2a (cm)	0.63	1.557	2.15	3.36
Surface area S = πa^2^ (cm^2^)	0.31	1.90	3.63	8.87
Well height h (cm)	1.115	1.798	1.700	1.180
r = h/2a	1.770	1.155	0.791	0.351
Number of cells *N_0_* (cells/well)	0.25 × 10^4^	1.25 × 10^4^	2.5 × 10^4^	5.0 × 10^4^
Initial cell density *N_0_*/*S* (cells/cm^2^)	8.0 × 10^4^	6.6 × 10^4^	6.9 × 10^4^	5.6 × 10^4^
Culture medium *v* (mL)	0.1	0.5	1.0	2.0
Medium height *l* = *v*/*S* (cm)	0.321	0.263	0.275	0.226

**Table 3:** The estimated parameters of 4-parameter sigmoid curve fitting and coefficient of determination (R^2^) on IL-6 release against UV dose.

Container	a	b	c (*D*_1/2_)	d	R^2^
3.5 cm dish	2.0	2.4	18.5	298.9	0.997
12-well plate	26.5	9.1	23.7	314.6	1.000
24-well plate	15.5	3.4	51.4	469.2	0.998
96-well plate	7.3	4.2	166.2	370.9	0.999

a: the bottom of the curve or lower plateau, b: slope factor, c: the concentration corresponding to the response midway between a and d. d: the upper plateau. The estimated c value represents the UV dose causing half the level of IL-6 release, defined as *D*_1/2_.

## Data Availability

The raw data supporting the conclusions of this article will be made available by the authors on request.

## Supplementary Materials

The following supporting information can be downloaded at: https://www.mdpi.com/article/10.3390/biotech13040044/s1, Supplementary Materials S1: a python code for fitting by a 4-parameter sigmoid curve; Supplementary Materials S2: a python code for calculating D′.

## Appendix A. Model Development

Consider a well placed in the ρψz-space with the center of the bottom surface at origin O (Figure A1a). Let the surface be S, where the dose received at each coordinate on S is denoted as $z_{E}\left(\rho ,\psi \right)$. The concept of the modeling is focusing on one coordinate on S and considering all rays converging at that point. Here, it should be noted that these rays passed through surface S′, which is the circle congruent to S on the z = h plane. UV dose D (mJ/cm^2^) was first multiplied by the reduction factor 10^−A^ and then averaged over the unit sphere centered at X to result in $z_{E}\left(\rho ,\psi \right)$. Subsequently, $z_{E}\left(\rho ,\psi \right)$ was weighted with the ratio between the solid angle subtended by S and 2π to yield $D^{\prime }$ (mJ/cm^2^). The integration range Ω in spherical coordinates is the projection of S′ onto the unit sphere. The intersection point of the circle O and the plane directed to ρ>0 is named Y (Figure A1a,b). The length of XY was defined as s. From the geometrical consideration (Figure A1c), s is expressed as follows:

(A1) $$s=\sqrt{a^{2}-\rho^{2}{sin}^{2}\phi }-\rho \cos\phi$$

Due to circular symmetry, D′ only depends on ρ, as follows:

(A2) $$\begin{array}{cc} D’ & =\frac{1}{S}\int_{S}z_{E}\left(\rho ,\psi \right)dS\\ & =\frac{1}{S}\int_{0}^{2\pi }\int_{0}^{a}z_{E}(\rho )d\rho d\psi \\ & =\frac{2\pi }{S}\int_{0}^{a}\rho z_{E}\left(\rho \right)d\rho \end{array}$$

where $z_{E}\left(\rho \right)$ is a function with ψ=0 in $z_{E}\left(\rho ,\psi \right)$. Assuming the wall of the well is impermeable to UVB, at point $X\left(\rho ,0\right)$, the spherical coordinates were set as to be placed with X as the center (Figure A1a). Let θ be the angle measured from the vertical direction, defined as the z’-axis, at point X, with UVB rays illuminated at an angle θ, where the range depends on h. The endpoints of the interval of θ are defined as $\theta \left(\phi \right)$. From geometrical considerations (Figure A1b):

(A3) $$\theta \left(\phi \right)=\cos^{-1}\left(\frac{h}{\sqrt{h^{2}+s^{2}}}\right)$$

Then, $z_{E}\left(\rho \right)$ can be expressed as follows:

(A4) $$z_{E}\left(\rho \right)=\frac{1}{2\pi }\int_{\Omega }D\times 10^{-A}d\Omega$$

The area element for this integration is calculated as sin⁡θdθdϕ (Figure A1a), and Equation (A4) can be expressed as follows:

(A5) $$z_{E}\left(\rho \right)=\frac{1}{2\pi }\int_{0}^{2\pi }\int_{0}^{\theta (\phi )}D\times 10^{-\frac{\varepsilon l}{\cos\theta }}\cdot \sin\theta d\theta d\phi$$

Noting that the optical length *l* is varied with angle of incidence θ, *l*/cosθ is used instead (Figure A1d). Finally, Equation (A2) is expressed as follows:

(A6) $$D^{\prime }=\frac{D}{S}\int_{0}^{a}\rho \int_{0}^{2\pi }\int_{0}^{\theta (\phi )}10^{-\frac{\varepsilon l}{\;\cos\theta }}\cdot \sin\theta d\theta d\phi d\rho$$

Generally, the integral in Equation (A6) cannot be solved analytically. Therefore, Python codes were developed to solve this, as detailed in Supplementary Materials S2.
