# Supplementary material for: Ultraviolet, Did the Cell See It from the Side or the Bottom? Assessment and Modeling of UV Effects on Cultured Cells Using the CL-1000 UV-Crosslinker
Source: BioTech (Basel). 2024 Oct 25;13(4):44. doi: 10.3390/biotech13040044 (PMC11587097; doi:10.3390/biotech13040044)
Supplement: Supplementary file 1 [file biotech-13-00044-s001.zip › biotech-3158068-supplementary.pdf]

BioTech

MDPI

Supplemental Material

Ultraviolet, Did the Cell See It from the Side or the Bottom? Assessment and Modeling of UV Effects on Cultured Cells Using the CL-1000 UV-Crosslinker

Takahiro Oyama, Kai Yanagihara, Anna Arai, Takanori Kamiya, Midori Oyama, Takashi Tanikawa, Takehiko Abe and Tomomi Hatanaka

Supplemental Material S1

```
import ipywidgets as widgets
from ipywidgets import VBox, HBox, FloatText, Checkbox, ColorPicker
from IPython.display import display
import pandas as pd
import matplotlib.pyplot as plt
from scipy.optimize import curve_fit
import numpy as np
import seaborn as sns
import os

# Update default plot settings
plt.rcParams.update({'font.size': 16, 'lines.markersize': 8, 'errorbar.capsize': 5})

# create_sigmoid 関数に Unit と Char_size を追加
# Create the sigmoid function with Unit and Char_size parameters added
def create_sigmoid(data, fit_color='r', scatter_color='b', scatter_size=50,
                  X_auto=True, X_lower=None, X_upper=None,
                  Y_auto=True, Y_lower=None, Y_upper=None, Y_interval=None,
                  Unit=True, Char_size=20):

    # 1. Read the Excel file
    excel_data = pd.ExcelFile(data)
    sheet_names = excel_data.sheet_names

    # 2. Create a list to record the estimated parameters
    params_list = []

    # Create output directory
    output_dir = os.path.splitext(data)[0]
    if not os.path.exists(output_dir):
        os.makedirs(output_dir)
```

```python
    # 3. Process each sheet and save each sheet's plot
    for i, sheet_name in enumerate(sheet_names):
        fig, ax = plt.subplots(1, 1, figsize=(6, 6))

        # Read the data from each sheet
        df = excel_data.parse(sheet_name)
        X = df['X']
        Y = df['Y']
        SE = df['SE']
        use = df['use']

        # Filter rows where the "use" column is 1
        X_filtered = X[use == 1]
        Y_filtered = Y[use == 1]

        # Define the function for curve fitting
        def fit_func(X, a, b, c, d):
            return d + (a - d) / (1 + (X / c) ** b)

        # Estimate parameters
        popt, pcov = curve_fit(fit_func, X_filtered, Y_filtered)
        Y_fit = fit_func(X_filtered, *popt)
        residuals = Y_filtered - Y_fit
        ss_res = np.sum(residuals**2)
        ss_tot = np.sum((Y_filtered - np.mean(Y_filtered))**2)
        r_squared = 1 - (ss_res / ss_tot)

        # Record the estimated parameters
        params_list.append({'Sheet': sheet_name, 'a': popt[0], 'b': popt[1], 'c':
popt[2], 'd': popt[3],
                            'a_error': np.sqrt(pcov[0, 0]), 'b_error': np.sqrt(pcov[1,
1]),
                            'c_error': np.sqrt(pcov[2, 2]), 'd_error': np.sqrt(pcov[3,
3]),
                            'R2': r_squared})


        # Plot all data
        ax.scatter(X, Y, color=scatter_color, s=scatter_size, label='Data (All)',
alpha=0.5)
        ax.errorbar(X, Y, yerr=SE, fmt='o', color=scatter_color, alpha=0.5)
```

```
        # Plot the fitted curve for filtered data
        X_fit = np.linspace(np.partition(X, 1)[1] / 2, X.max() * 2, 1000)
        Y_fit = fit_func(X_fit, *popt)
        ax.plot(X_fit, Y_fit, color=fit_color, label='Fit (Filtered)')

        # Set axis properties
        ax.set_xscale('log')
        if X_auto:
            ax.set_xlim(np.partition(X, 1)[1] / 2, X.max() * 2)
        else:
            ax.set_xlim(X_lower, X_upper)

        if not Y_auto:
            ax.set_ylim(Y_lower, Y_upper)
            ax.yaxis.set_major_locator(plt.MultipleLocator(Y_interval))

        # Set minor ticks for the x-axis
        ax.tick_params(axis='x', which='both', direction='in')

        sns.despine()
        ax.tick_params(axis='both', direction='in')

        # If Unit is False, remove axis numbers
        if not Unit:
            ax.set_xticklabels([])
            ax.set_yticklabels([])

        # If Unit is True, change font size
        else:
            ax.tick_params(axis='both', labelsize=Char_size)

        # Save the figure
        fig.savefig(os.path.join(output_dir, sheet_name + '.png'))
        plt.close(fig)

    # Summarize parameters into a DataFrame
    params_df = pd.DataFrame(params_list)

    # Save the DataFrame to an Excel file
    params_df.to_excel(os.path.join(output_dir, 'params_summary_filtered.xlsx'),
index=False)

# Update the upload_and_process_file function to get file name from metadata
def upload_and_process_file(change):
```

```
    uploaded_file = change['new']
    if uploaded_file:
        file_info = list(uploaded_file.values())[0]
        file_content = file_info['content']

        # Debugging print statement
        print(file_info)   # For debugging
        file_name = file_info['metadata']['name'] # Get file name from metadata
        with open(f"/content/{file_name}", "wb") as f:
            f.write(file_content)
        create_sigmoid(f"/content/{file_name}", fit_color=fit_color.value,
scatter_color=scatter_color.value, scatter_size=scatter_size.value,
                     X_auto=X_auto.value, X_lower=X_lower.value, X_upper=X_upper.value,
                     Y_auto=Y_auto.value, Y_lower=Y_lower.value, Y_upper=Y_upper.value,
Y_interval=Y_interval.value,
                     Unit=Unit.value, Char_size=Char_size.value)

# Function to create the GUI
def create_gui():
    global fit_color, scatter_color, scatter_size, X_auto, X_lower, X_upper, Y_auto,
Y_lower, Y_upper, Y_interval, Unit, Char_size

    fit_color = ColorPicker(description='Fit Color', value='red')
    scatter_color = ColorPicker(description='Scatter Color', value='blue')
    scatter_size = FloatText(description='Scatter Size', value=50)
    X_auto = Checkbox(description='X Axis Auto', value=True)
    X_lower = FloatText(description='X Lower', value=1, disabled=True)
    X_upper = FloatText(description='X Upper', value=100, disabled=True)
    Y_auto = Checkbox(description='Y Axis Auto', value=True)
    Y_lower = FloatText(description='Y Lower', value=0, disabled=True)
    Y_upper = FloatText(description='Y Upper', value=1.5, disabled=True)
    Y_interval = FloatText(description='Y Interval', value=0.1, disabled=True)
    Unit = Checkbox(description='Unit', value=True)
    Char_size = FloatText(description='Char Size', value=20)

    X_auto.observe(lambda change: set_disabled_state(change, X_lower, X_upper),
names='value')
    Y_auto.observe(lambda change: set_disabled_state(change, Y_lower, Y_upper,
Y_interval), names='value')

    upload_button = widgets.FileUpload(description="Upload Excel File")
    upload_button.observe(upload_and_process_file, names='value')
    output = widgets.Output()
```

```
    ui = VBox([
        HBox([fit_color, scatter_color, scatter_size]),
        HBox([X_auto, X_lower, X_upper]),
        HBox([Y_auto, Y_lower, Y_upper, Y_interval]),
        HBox([Unit, Char_size]),
        upload_button,
        output
    ])

    display(ui)

def set_disabled_state(change, *args):
    for widget in args:
        widget.disabled = change.new

# Start the GUI
create_gui()
```

****

This code works in Google Collaboratory. This application reads data from an Excel file, fits a sigmoid curve to the data on each sheet, saves the estimated parameters and R2 value of the fit in an Excel file, and visualizes the results as graphs.
The input Excel format is like

X	Y	SE	use
0	7.343704	9.065442	1
5	4.990555	3.542676	1
10	61.49709	27.92398	1
20	162.6343	56.73978	1
50	274.781	118.5767	1
100	74.72979	27.66278	0

Execute the code cell to display the interface. Click the "Upload Excel File" button and select the Excel file you want to fit curves to.

Supplemental Material S2

```
import numpy as np
import pandas as pd

# Set constants
# Define the range of phi values from 0 to 2*pi (used in integration)
phi_values = np.linspace(0, 2 * np.pi, 100)

# Define epsilon, which is the relative molar extinction coefficient for HuMedia
epsilon = 0.1695

# Set values for parameters 'a', 'h', 'l' and 'D'
# 'a' is a characteristic radius, 'h' is a height, 'l' is a length derived from volume,
and 'D' is a set of diffusion coefficients
a_values = [0.315, 0.779, 1.075, 1.680]
h_values = [1.115, 1.798, 1.70, 1.18]
l_values = [0.321, 0.263, 0.275, 0.226]  # Derived from volume: l = v/S = v/πa^2
D_values = [0, 2, 5, 10, 20, 30, 50, 100, 200, 500]

# Define helper functions
# Function to calculate 's', based on phi, x, and a
def s_calculation(phi, x, a):
    return np.sqrt(a**2 - x**2 * np.sin(phi)**2) - x * np.cos(phi)

# Function to calculate 'alpha' (or theta in the manuscript), based on phi, x, a, and h
def alpha(phi, x, a, h):
    s_val = s_calculation(phi, x, a)
    return np.arccos(h / np.sqrt(h**2 + s_val**2))

# Initialize list to store final results
results = []

# Loop through each D value (diffusion coefficients)
for D in D_values:
    D_prime_scenarios = []

    # Loop through each scenario (4 cases with different a, h, l values)
    for i in range(4):
        a = a_values[i]
        h = h_values[i]
        l = l_values[i]

        # Set the range of x values (rho in the manuscript) for integration
        x_values = np.arange(0, a, 0.01)
```

```
        integral_2_values = []

        # Loop through each x value to calculate integral_2
        for x in x_values:
            integral_1_values = []

            # Loop through each phi value for the first integral
            for phi in phi_values:
                s_val = s_calculation(phi, x, a)
                alpha_value = alpha(phi, x, a, h)  # Calculate alpha (or theta) for each
phi

                # Generate a range of theta values for integration (from 0 to alpha)
                theta_values = np.linspace(0, alpha_value, 100)
                cos_theta = np.cos(theta_values)
                sin_theta = np.sin(theta_values)

                # Calculate the expression to be integrated, which involves D, epsilon,
l, cos_theta, and sin_theta
                expression = D * 10**(-epsilon * l / cos_theta) * sin_theta

                # Perform numerical integration over theta values using the trapezoidal
method
                integral_1 = np.trapz(expression, theta_values)
                integral_1_values.append(integral_1)

            # After looping through phi, perform the second integral over phi values
            integral_1_values = np.array(integral_1_values)
            integral_2 = np.trapz(integral_1_values, phi_values)
            integral_2_values.append(integral_2)

        # Now integrate over x to compute z_E_x, which is the final result of this step
        integral_2_values = np.array(integral_2_values)
        z_E_x = np.trapz(integral_2_values * x_values, x_values)

        # Calculate D_prime for the current scenario
        D_prime = z_E_x / ((np.pi) * (a**2))
        D_prime_scenarios.append(D_prime)

    # Append results for the current D value and all scenarios
    results.append([D] + D_prime_scenarios)

# Create a DataFrame to store and display the results
columns = ['D'] + [f'Scenario {i+1} D_prime' for i in range(4)]
```

```
df = pd.DataFrame(results, columns=columns)

# Print the resulting DataFrame
print(df)
```
